# Long-term effects of group exercise intervention on maximal step-up height in middle-aged female primary care patients with obesity and other cardio-metabolic risk factors

**DOI:** 10.1186/s13102-020-00161-4

**Published:** 2020-03-16

**Authors:** Lillemor A. Nyberg, Carl Johan Sundberg, Per Wändell, Jan Kowalski, Mai-Lis Hellénius

**Affiliations:** 1 Department of Medicine and School of Health Sciences, Örebro University, 70182 Örebro, Sweden; 2 Division of Family Medicine and Primary Care, Department of Neurobiology, Care Sciences and Society, Karolinska Institutet, Stockholm, Sweden; 3 Karolina Primary Health Care Centre, Karlskoga, Region Örebro County Sweden; 4 Department of Physiology and Pharmacology, Karolinska Institutet, Stockholm, Sweden; 5 Department of Clinical Science, Intervention and Technology, Karolinska Institutet, Stockholm, Sweden; 6 Department of Medicine, Karolinska Institutet, Stockholm, Sweden

**Keywords:** Muscle strength, Step-up height, Exercise, Primary care, Rehabilitation, Female

## Abstract

**Background:**

Low physical performance is a predictor of morbidity and mortality. This study looks at long-term effects of an exercise intervention on maximal step-up height (MSH) in individuals with low physical function. Factors associated with changes in MSH was studied.

**Methods:**

Female patients (*n* = 101), mean (SD) age of 52 (11) years, were recruited for a 3-month group exercise intervention including 2–3 sessions/week of mixed aerobic fitness and strength training. MSH, weight, body mass index (BMI), waist circumference, maximal oxygen consumption (VO_2_-max), self-reported health (SF-36) and physical activity (PA) were measured at baseline (T0), after 3 months (T1) and after 14–30 (mean 22) months (T2). Relationships between changes in MSH (cm) and age, baseline MSH, time to follow-up, changes in anthropometric measurements, VO_2_-max, SF-36 and PA were studied with regression analyses.

**Results:**

MSH, significantly, increased from T0 to T1, 27.2 (5.7) to 29.0 (5.5) cm and decreased to 25.2 (5.5) cm at T2. Time to follow-up (B = − 0.42, *p* < 0.001) and change in BMI (B = − 0.29, *p* = 0.012) correlated significantly to changes in MSH. Waist circumference, VO_2_-max, PF and exercise/physical activity levels were significantly improved at T2, while BMI did not change. In a univariate logistic regression model, maintenance of MSH correlated to the extent of mixed training (OR 3.33, 95% CI 1.25–8.89). In a multivariate logistic regression model adjusted for important factors the correlation was not significant. However, MSH was significantly higher in individuals participating in 2–3 session per week compared to one session.

**Conclusions:**

A 3-month group exercise intervention increased MSH, improved fitness, decreased risk in female patients with elevated cardio-metabolic risk. After an average of 22 months MSH was reduced while positive effects remained for waist circumference, VO_2_-max, physical function and physical activity. However, regular group exercise 2–3 times per week with mixed aerobic fitness and strength training was associated with maintenance of MSH in a subgroup of patients. We suggest that such an intervention including regular support from healthcare professionals is a successful approach for maintaining improved leg-muscle strength among primary care patients.

**Trial registration:**

ISRCTN21220201 September 18, 2019, retrospectively registered.

## Background

A large number of studies have shown a strong correlation between physical activity (PA) and health [1, 2] and both physical activity and aerobic fitness are independently associated with lower cardiovascular risk [3, 4]. Studies show a strong dose-response relationship between the degree of PA and premature death [5–8], cardiovascular disease, type 2 diabetes [9], osteoporosis, colon and breast cancer, asthma/COPD, depression [10], anxiety [11], dementia and symptomatic osteoarthritis [12]. Studies have also shown a strong correlation between weak muscular strength and a high prevalence of obesity and metabolic syndrome [13], premature death [14–16] and cancer [17]. A large population-based study published 2015 and conducted over four years with nearly 140,000 individuals—58% women with median age 50 (42–58) years from 17 countries and with varying income and sociocultural background—showed that grip strength was a stronger predictor of all-cause mortality and cardiovascular mortality than systolic blood pressure [18].

New research shows that dietary patterns are important for promoting skeletal muscle hypertrophy [19, 20], systemic anti-inflammation and for reducing metabolic risk in older women [21], independently of time spent in moderate-to-vigorous PA [22], and that the evaluation of functional capacity can help to determine the degree of physical decline in persons with metabolic syndrome [13]. PA had similar effect on employment in patients on sick leave due to depression as usual treatment [23]. The effect of resistance training in relation to depression has not been completely determined yet [24]. To optimize prevention and treatment of common diseases and disorders with PA, aerobic and resistance training, knowledge about the patient’s psychological symptoms, physical function, leg-muscle strength [25] and eating behaviors [22] is important. Also importance of enjoyment promoting exercise have been studied [26].

Therefore, a standardized method for testing leg-muscle strength and leg function in each leg to assess an individual’s maximal step-up height (MSH)—i.e. the mean step-up height of left and right leg—has earlier been developed [27]. The standardized maximal step-up test (MST) was tested for repeatability and validity in a working middle-aged population (50% women). MSH was positively correlated to thigh-muscle strength measured with an isokinetic Quadriceps strength test (Biodex) and self-reported physical function measured with SF-36, PF items. Those who stepped higher than 32 cm reported no impaired physical function [27].

Previously, we have also investigated the short-term effects of a 3-month lifestyle intervention with group exercise program, on MSH and correlations between cardio-metabolic risk factors and MSH, as well as the relationship between changes of MSH and changes in cardio-metabolic factors, in a population of female primary care patients [28]. Variations to changes in MSH were explained by changes in waist circumference and physical function (SF-36, PF items), regardless of age and changes in VO_2_-max. A maximal step-up height below 24 cm identified patients with self-reported severe limitations in physical function. MSH correlated with training intensity [28].

The maximal step-up test [27] has also been mentioned as a test of interest in future research on knee and hip osteoarthritis [29].

The main aim of this study was to investigate—in a female patient population with common diseases, elevated cardio-metabolic risk, musculoskeletal pain and reduced capacity for work—the long-term effects of a 3-month group exercise programme on MSH, cardio-metabolic risk factors and physical functioning. Furthermore, we studied factors associated with long-term effects on MSH in the whole study population, in subgroups of patients of different ages, as well as in subgroups with high, medium or low maintenance of MSH. Inter-examiner repeatability test was included in this study. We have hypothesized that simple tests of patient’s leg strength and leg function in everyday clinical practice, could be useful.

## Methods

### Design

Interventional study without control group. Trial setting is GP practices and trial type is treatment. Primary and secondary measurements were assessed at baseline (T0), after 3 months (T1) directly after the intervention, and at the long-term follow-up after mean 22 months (T2). Primary outcome measure: maximal step-up height (MSH). Secondary outcome measures: age, height, bodyweight, body mass index (BMI), waist circumference, aerobic fitness (VO2-max), self-reported physical activity (PA) and health-related quality of life (SF-36 scores), the subscale of physical function (PF). Inter-examiner repeatability test at T2.

### Subjects

Out of 214 female patients consecutively referred from primary health care, 178 attended a first test (T0) and 156 participated in a 3-month group exercise intervention program and took part in a second assessment (T1) [28]. Out of these 156 patients, 114 were randomly invited for a third assessment (T2) out of whom 101 agreed to participate. No significant difference in baseline measurements between the 101 participating patients and remaining 77 were found regarding anthropometry, education, sick leave, self-reported pain and/or reduced physical function. However, age differed significantly, with a higher mean age in the 101 participants compared to the 77 who did not participate in T2 (mean 52 years vs. 47 years, *p* = 0.003).

### Intervention with group training

The 3-month group exercise intervention, tailored to participants’ needs, has previously been described in detail [28].

In brief, 3 weekly training sessions—chosen from different types of group exercise—were agreed with each patient when they began the program. The training was supervised by medically trained and experienced coaches and conducted at local gyms, the municipal swimming pool, on outdoor tracks and in school facilities. At each training session, coaches explained the intensity of the exercise, controlled, for instance, by the pace of music or by the duration of sessions of Nordic walking. The different levels of session intensity—classified as very light, light, or moderate—helped the coach to guide the patient to start at a suitable level depending to baseline tests, to minimise any cardiovascular risks, or acute or overuse injuries. The patients were trained to use the 6–20 Borg RPE Scale [30] to record perceived intensity. Level 13–15 was recommended as a goal at each training session once they individually had received the go-ahead from the coach. Healthy lifestyle to promote positive effects of the training on especially muscle mass and strength was recommended by the coach during intervention; no nicotine two hours before and after the exercise, and adequate food intake more than two hours before exercise and directly after training, was recommended.

At the end of the 3-month program, patients—together with their coaches—planned how to continue with similar activities after the program had ended**.** Both during and after the 3-month intervention period patients were offered standard primary care, including customary follow-up appointments.

### Measurements

#### Physical examination

Body weight was measured in light clothing without shoes to the nearest 0.1 kg using electronic scales (Seca Delta model 707). Height was measured without shoes to the nearest 0.5 cm using a measuring stick fixed to the wall. Body mass index (BMI) was calculated according to the standard formula (kg·m^− 2^). Waist circumference (cm) was measured at the level of the umbilicus according to standard practice, Table 1.

**Table 1:** Characteristics (mean (SD)) of study population at baseline and changes at follow-up

***n*** = 101	T0	T0–T1	T0–T2
^a^Age	52.4(11.4)	0.3(0.2)	1.8(0.4)
^b^Body composition:			
Height	164.0(6.3)	N/A	N/A
Weight	77.7(16.5)	−0.9(3.7)*	− 0.2(5.7)
BMI	28.9(5.9)	−0.3(1.3)*	−0.1(2.1)
Waist circumference, ^c^*n* = 99	94.8(15.1)	0.0(0.1)	−2.4(0.6)***
^d^**Exercise/PA, 5 levels,** 3*n* = 94 (range)	2.7(1.2) (1–5)		0.6(1.2)*** (−3 − + 3)

**Time points: T0** = baseline measurements in a lifestyle and exercise intervention programme in female primary care patients (*n* = 101), **T0**–**T1** = change at follow-up after 3 months of mostly mixed aerobic fitness and strength training, **T0**–**T2 =** change at 14–30-month follow-up with self-reported exercise/ physical activity registered from diaries covering 3 months before **T2**, mostly brisk walking. ^a^Age (years). ^b^Body composition; height (cm), weight (kg), BMI i.e. body mass index and waist circumference (cm). ^c^n i.e. number analysed per protocol. ^d^Exercise/physical activity for fitness, health and wellness; 1 = never, 2 = now and then, 3 = 1–2 times/week, 4 = 3–5 times/week and 5 = more than 5 times/week. Standard deviation = (SD). * Denotes p-level < 0.05; *** denotes p-level < 0.001

#### Diagnosis, work status and capacity for work

A standardized and structured protocol was used by GPs to refer patients to the intervention program. It included common diseases, joint pain or discomfort at 14 specified localizations, and a record of medication. The capacity for work, prevalence of sick leave, disability pension and job seeking status were also recorded in the protocol, Table 2.

**Table 2:** Study population characteristics (mean (SD)) for self-reported physical and mental function, physical limitations and work status

^a^SF-36 score mean (SD)	T0	T0 — T1	T0 — T2
*Physical*			
PF-Physical Function	67.1(17.8)	5.9(14.6) ***	5.4(17.2)**
RP-Role Limitation Physical	35.4(39.1)	6.4(35.8)	12.6(45.4)**
BP-Bodily pain	39.7(20.8)	9.3(17.5)***	6.8(22.6)**
GH-General Health	45.2(19.0)	7.6(17.3)***	7.4(20.5)**
*Mental*			
VT-Vitality	36.2(22.0)	13.5(20.6)***	10.5(24.5)***
SF-Social Function	65.6(27.0)	9.4(23.4)***	6.4(31.1)*
RE-Role Limitation Emotional	53.5(46.0)	8.6(41.8)*	11.9(46.3)*
MH-Mental health	63.8(22.7)	5.5(19.6)**	6.8(23.9)**
^b^PF, items limitation Severely (%) / somewhat (%) / not (%)	T0	T1	T2
3b	15 / 63 / 23	7 / 61 / 33	15 / 50 / 36
3d	18 / 45 / 38	8 / 49 / 44	9 / 48 / 44
3f	19 / 43 / 39	13 / 49 / 39	19 / 36 / 46
3 g	17 / 35 / 49	8 / 27 / 66	10 / 27 / 64
without any item limitations	7 (7%)	17 (17%)	21 (21%)
≥ 1 item severely limited	38 (38%)	25 (25%)	35 (35%)
^c^Work status, % / % / %	T0		T0 — T2
working/ applying for job/ studying (≥ 50%)	62 / 8 / 3		−11 / 4 / -2
sick leave (%), 25 / 50 / 75 / 100	5 / 12 / 1 / 31		−3 / -7 / 1 / -13
sickness benefit/ sickness pension	3 / 9		3 / 2
age pension/ housewife (100%)	14 / 2		4 / 0

**Time points: T0** = baseline values in a lifestyle and exercise intervention program in female primary care patients (*n* = 101), **T1** = follow-up after 3 months with mostly mixed aerobic fitness and strength training, **T2 =** 14–30-month follow-up after 3 months with self-reported exercise/ physical activity registered from diaries, mostly brisk walking. ^a^SF-36, with eight domains of health status, and summary scores of physical and mental components 0–100, with higher scores representing a better health status score. ^b^PF (SF-36) item 3b, 3d, 3f, 3 g; with per cent of patients severely limited/ somewhat limited/ not limited. 3b: activities such as lifting a table, vacuum cleaning, walking in the forest and gardening; 3d: activities such as climbing several flights of stairs; 3f: activities such as bending, kneeling or stooping; and 3 g: activities such as walking more than 2 km. ^c^Capacity for work, full and part-time reported, categorised as working/ applying for job/ studying when ≥50% and as sickness benefit/ sickness pension if > 50%. Work status is presented as proportions of the study population (%). Standard deviation = (SD). * Denotes p-level < 0.05; ** denotes p-level < 0.01; *** denotes p-level < 0.001

#### Physical function score and any limitation score

The Swedish version of SF-36 [31] assessing health-related quality of life was filled out by patients in advance of admission to the program. Where needed, assistance was given at the time of the first visit. All raw scores were transposed onto scores on a weighted 0–100 scale, higher scores representing better health status [32]. The subscale PF was extracted for further item analyses; the items describing doing moderate activities (3b), climbing several flights of stairs (3d), bending, kneeling or stooping (3f) and walking more than 2 km (3 g). We analysed: i) all eight scales (0–100); ii) any limitation score in the PF items 3b, 3d, 3f and 3 g, respectively, ranging from 1 to 3 (1 = severely limited, 2 = somewhat limited, 3 = no limitation); and iii) the sum of 3b, 3d, 3f and 3 g ranging from 4 to 12 (from 4 = severely limited in all items to 12 = no limitation in any of the four items), Table 2.

#### Physical capacity, testing procedures and equipment

Maximal step-up height (MSH) (cm) was assessed by the standardized maximal step-up test (MST) on each leg. MST has previously been described including tests of validity and repeatability [27]. The intra-examiner test-retest (one week between occasions) showed MSH repeatability of 6.9 and 5.9 cm for right and left leg, respectively. Levels are pre-set at 3 cm apart on a tailor-made device called the step-up box. If a patient tried and failed at the lowest MSH level with both legs, MSH mean value for statistical analysis was interpreted as zero. If one leg failed and the other leg managed 18 cm, the mean MSH registered was 9 cm. A missing value was registered for each leg when a patient did not try, and no mean MSH was calculated. The MST method includes information about how to instruct and encourage the patient during the test. In brief, the tester demonstrates how the standardized MST is performed and instructs patients to step up without assistance from the foot that remains on the floor and without support from handrails. Patients are allowed three attempts at the highest level for each leg with verbal encouragement given. The suggested goal for each patient is to manage MSH with 90 degrees at hip and knee while in the starting position.

The VO2-max was estimated as described by Åstrand [33], one of the most commonly used submaximal exercise tests. In the original article reported validation was r = 0.78 and the coefficient of variation (CV) was 15% for a mixed sample of subjects. In new research, published after our study was conducted, the validity correlation coefficient for the Ekblom-Bak submaximal exercise test [34] was r = 0.91 and CV was 9,3% compared to results from Åstrands test in that study, r = 0.68 and CV 18.1% From each participant’s individual heart rate response to a given sub-maximal workload (i.e. 50–150 W, depending on the participant’s weight and self-reported physical activity) using a bicycle ergometer (Monark Exercise AB, Vansbro, Sweden), oxygen consumption was recorded at a steady-state with a heart rate 120–150 beats·min^− 1^, and quantified as peak absolute oxygen consumption per minute (L·min^− 1^) and per kg body weight per minute (mL·kg^− 1^·min^− 1^).

### Self-reported exercise and PA levels

At baseline (T0) and at the third assessment (T2), exercise and PA were assessed using a validated questionnaire common in occupational health care in Sweden [35]. The PA question included a definition of exercise, i.e. to allocates time for exercise in order to maintain or improve fitness, health and wellness, and one of five levels were registered, se Table 2 [35].

#### Training and physical activity measurements

Leisure-time PA or sedentary behaviour was not registered during the 3-month group exercise intervention. Patients filled in their own exercise diary at each training session and coaches checked and signed the diary. The self-selected workouts were evaluated in groups of i) mixed aerobic fitness and strength training, ii) aerobic fitness training, and iii) strength training. The dose in times per week (t.p.w), minutes per week (m.p.w) and intensity (6–20 Borg RPE Scale) during the 60-min workout sessions were recorded. The number of dance therapy and Qigong sessions (replacing other types of workouts and registered as mostly aerobic fitness and strength training, respectively)—often chosen because of fear of movement or to improve balance and coordination—were registered and included in total exercise. Exercise and PA levels were estimated from both patient diaries from 3 months preceding the long-term follow-up and also from questionnaires at long-term follow-up [35]. An effort equivalent to at least 30 min of brisk walking corresponding to level light to somewhat hard 11–13 Borg RPE Scale was required for registration as a session.

### Statistics

Maximal step-up height was used as the primary end-point. MSH was calculated as mean step-up height of left and right leg. Results from two measurements by two test leaders, with 30-min interval, at the long-term follow-up was analysed with help of ANOVA to calculate repeatability [36, 37]. We expect 95% of differences between paired observations, i.e. test leaders, to be less than this definition of a repeatability adopted.

MSH and cardio-metabolic main variables and their changes from baseline to 14–30-month follow-up after the 3-month intervention program were analysed using the students T-test. Logistic regression analysis was performed to explore subjects with maintained MSH as outcome (maintained or increased MSH vs. decreased MSH). Mixed aerobic fitness and strength training sessions were dichotomized into high (> 21 sessions during the 3-month training period) or low (≤ 21 sessions). Age (years), time to follow-up (months) and MSH (cm) at baseline were included as covariates, as these were found to be significant in univariate analyses. Results are presented as odds ratio (OR) with 95% confidence interval (95% CI). Model specification was tested by Pearson’s and Hosmer-Lemeshow’s tests.

To describe differences between the two subgroups with the highest MSH maintenance (increased or maintained) and the subgroup with the lowest MSH maintenance (decreased), the Mann-Whitney U-test, 2*1-sided exact p test was used. Furthermore, the data was also split in tertiles of age (two outliers, 81 and 83 years old at baseline, were added to the oldest group). All tests were two-sided and performed on the 0.05 level of significance. No adjustments for multiplicity were done as this is an exploratory study. Overall, we can expect 1/20 statistical significant results to be caused by random for analysis in independent variables. Statistica v 12.0, StatSoft Inc., Tulsa, United States, was used for statistical analyses.

## Results

### Inter-examiner repeatability test at long-term follow-up

The inter-examiner (minimum 30 min) repeatability was 3.94, i.e. in 95% of the mean MSH the right and left leg assessments. A variation up to 4 cm will be expected between trained examiners following the standardization including not allowing a kick-off with the floor foot [27].

### Characteristics of the study population at baseline

Some characteristics of the study population are presented in Table 1 and Table 2. The mean BMI and mean waist circumference at baseline described that a majority of the study population was overweight and abdominal obesity was common. Mean (SD) VO_2_-max was 27.5 (7.3) mL·kg^− 1^·min^− 1^ and MSH was 27.2 (5.7) cm.

Furthermore, 74% were cohabiting, 20% were smokers, 18% of participants reported having completed elementary school as their highest educational qualification, 46% upper secondary school, and 37% degree level at university or college. The exercise habits before the age of twenty varied considerably: 2% reported being exempt from physical education (PE) class at school, 38% reported no training other than PE, 36% reported exercising through non-competitive participation in a ball sport, 19% did participate in both exercise and competitions, and 5% were serious participants in athletic competitions.

### Changes in exercise and physical activity, SF-36 and capacity for work and sick leave

As the long-term follow-up (the third assessment, T2) was conducted across three months, whereas participants had been referred to the study consecutively over a longer period of time, we obtained a time range between T0 and T2 of 14–30 months (mean 22 months).

Significant increases in self-reported exercise/PA five levels onto mean 22-month follow-up were analyzed, see Table 1. The main activity reported from patients’ diaries at T2 was brisk walking consisting of ≥30 min per day on an average of five days of the week. A substantial decline in group exercise was reported 3 months before T2 compared to T1, see Additional file 1.

Significant improvements in SF-36 scores were seen in all scales at both follow-ups as seen in Table 2. From SF-36 the items 3b, 3d, 3f and 3 g were evaluated separately and increased ability were seen in three of four of these physical functions, the limitations for knee bending (3f) did not significantly change by the two follow-ups. Compared to baseline there were some improvements concerning great limitations for “bending knees” at the 3-month follow-up occasion for 6/19 patients, but this change did not persist at long-term follow-up, see Table 2.

The proportion of patients with capacity to work—including workers, job seekers and students— increased from 33 to 58% until T2. The mean degree of sick leave—as a share of 1.0 (SD)—at T0 and T2 for the subgroup with highest maintenance of MSH (*n* = 27) were 0.45 (0.46) and 0.21 (0.41) (*p* = 0.002), respectively. These degrees were compared to the change in degree of sick leave from T0 to T2, 0.48 (0.46) to 0.28 (0.44) (*p* = 0.121) for the group with lowest MSH maintenance (*n* = 22).

### Changes in MSH and metabolic risk factors after group exercise intervention

**Fig. 1:**
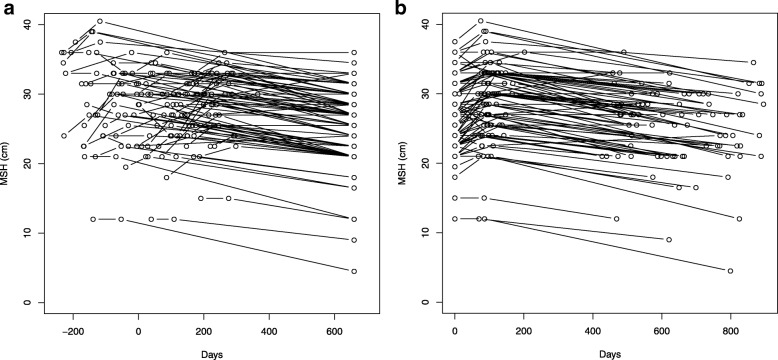
Maximal step-up height changes after a 3-month exercise intervention. **a** Maximal step-up height (MSH), i.e. the mean step-up height of left and right leg, for consecutively enlisted female patients referred to a 3-month intervention program of group training in primary health care. MSH from patients all three tests were plotted along a time axis, and the baseline measurement was put in relation to the start of the intervention project. A large spread of MSH values could be seen along both axes and high MSH was more often measured early on in intervention. **b** Maximal step-up height, i.e. the mean step-up height of left and right leg, for consecutively enlisted female patients referred to a 3-month intervention program of group training in primary health care. When all patients’ baseline measurements were plotted at the same place at the time axis, the significant positive change in MSH during the 3-month intervention program became apparent, as did the decline up until 14–30-month follow-up.

MSH values from all three test occasions were plotted along a time axis and a large distribution of MSH values could be seen at baseline as seen in Fig. 1a. When we placed three measurements at three places along a time axis, the positive change in MSH during the 3-month group exercise program was more clearly visualized, as was the—often parallel—decreases in MSH up until the long-term follow-up as shown in Fig. 1b.

Significantly, lower weight and BMI were seen at the 3-month follow-up, but there were no change in these variables at the long-term follow-up as seen in Table 1. Waist circumference was not changed at 3 months but was significantly lower, − 2.4 cm, compared to baseline at the long-term follow-up, Table 1 and Table 3. Significantly increased VO_2_-max at both follow-ups and increased MSH at 3-month follow-up and decreased MSH at long-term follow-up, were noted (Table 3).

**Table 3:** Baseline levels and changes (mean (SD)) after 3 and 14–30 months in main outcomes

T0	T0 — T1		T0 — T2
Total group			
(*n* = 101)			
Age	52.4(11.4)	+ 0.3(0.2)***	+ 1.8 (0.4) ***
MSH	27.2(5.7)	+ 1.5(2.2) ***	−2.4 (3.0) ***
Waist cm	94.8(15.1	+ 0.0(4.1)	− 2.4(6.6) ***
VO2- max	27.5(7.3)	+ 5.4(5.9) ***	+ 1.9(5.6) **
MSH subgroups			
MSH **a** (*n* = 27)			
age	48.9(13.3)		+ 1.6(0.3) ***
MSH	26.7(4.1)	+ 2.2(2.3) ***	+ 0.9(1.7) *
waist cm	94.6(13.9)	−0.5(6.1)	−4.7(8.7) *
VO2- max	28.3(5.8)	+ 7.3(6.6) ***	+ 4.7(6.0) **
MSH **b** (*n* = 40)			
age	55.4(9.1)		+ 1.8(0.4) ***
MSH	27.1(5.4)	+ 1,7(1.9) ***	−2.3(0.8) ***
waist cm	95.7(14.0)	+ 0.2(3.0)	−2.1(5.7) *
VO2- max	27,1(8.6)	+ 3.4(4.3) ***	+ 0.5(4.7)
MSH **c** (*n* = 22)			
age	50.4(10.5)		+ 2.1(0.3) ***
MSH	29.6(6.0)	−0.2(1.9)	−6.6(1.4) ***
waist cm	91.1(15.0)	+ 0.8(3.1)	+ 0.4(4.6)
VO2- max	27.5(5.8)	+ 7.9(6.3) ***	+ 2.1(6.2)
Age subgroups			
Age **a** (*n* = 32)			
age	39.6(5.6)		+ 1.9(0.4) ***
MSH	31,2(3.8)	+ 1.5(2.7) **	−2.1(3.9) **
waist cm	89.7(14.9)	+ 0.2(4.7)	−2.8(7.8)
VO2- max	33,0(6.5)	+ 6.1(6.1) ***	+ 3.6(6.3) **
Age b (*n* = 31)			
age	52.9(2.6)		+ 1.8(0.4) ***
MSH	26.2(4.7)	+ 1.4(1.6) ***	−2.2(2.4) ***
waist cm	96,7(14.3)	−0.3(3.4)	−2.5(5.9) *
VO2- max	23.8(5.3)	+ 5.2(6.5) ***	+ 1.1(5.2)
Age c (*n* = 35)			
age	64.2(6.5)		+ 1.8(0.3) ***
MSH	24,5(5.4)	+ 1.6(2.2) ***	−2.9(2.7) ***
waist cm	97.7(15.3)	+ 0.2(4.2)	−1.8(6.2)
VO2- max	25.9(6.0)	+ 4.6(5.0) **	+ 0.5(4.9)

Time points; T0 = baseline, T1 = follow-up after 3 months on the intervention program and T2 = follow-up after 14–30 months. Analyzed intention to treat. MSH subgroups according to level of maintenance; MSH a = highest, MSH b = medium and MSH c = lowest. * = *p* < 0,05, ** = < 0,01, *** = < 0,001

We found no significant seasonal differences in mean MSH at T0 and T1 or MSH changes from T0 to T1 assessments between seasons.

### Other subgroup analyses of MSH and changes of MSH at follow-ups

When the whole group was divided into tertiles by age at T0 there were differences in MSH, waist circumference and VO_2_-max between groups. The middle age group had measurements closer to the oldest group as seen in Table 3. Furthermore, other subgroup analyses presented in Additional file 2 show that obese patients with type 2 diabetes had a steeper MSH decline than obese patients without diabetes after the 3-month intervention program. A steeper decline in MSH was also found for patients with only hip pain compared to those with combined hip/knee or only knee pain. Additional file 2 also presents supervised group exercise intensity, neck and shoulder pain and mental diseases, the subgroup explorative analysis of mean MSH and MSH changes between T0 and T1, and T0 and T2, respectively.

### MSH correlations and regression analysis

Univariate correlations between main variables and the changes from baseline to follow-up after 14–30 months are shown in Table 4. The decline in MSH correlated to follow-up time and changes in weight, BMI and waist circumference. Reduction in waist circumference was significantly correlated with the total number of registered exercise sessions before long-term follow-up, see Table 4.

**Table 4:** Correlations between changes in mean maximal step-up height (MSH) and changes in other variables

Variable	1 Follow-up time	2 Diff weight	3 Diff BMI	4 Diff waist	5 Diff MSH	6 Total exercise
**1**	1.00					
**2**	0.11	1.00				
**3**	0.10	1.00*	1.00			
**4**	0.23*	0.66*	0.66*	1.00		
**5**	−0.48*	−0.37*	−0.36*	−0.27*	1.00	
**6**	−0.12	−0.15	− 0.14	−0.32*	0.08	1.00

Two models of multivariate regression analysis were performed: the first model is recorded in Table 5 (R^2^ = 0.620) with MSH at 14–30 months as dependent variable; and second model is recorded in Table 6 (R^2^ = 0.426) with the change in MSH from baseline to long-term follow-up as dependent variable. The cardio-metabolic risk variables and the follow-up time are included in the models.

**Table 5:** Regression model with mean maximal step-up height (^a^MSH) as dependent variable at 22-month follow-up

Variables	Beta (SE)	Beta (SE)	t (37)	p-level
intercept		30.96(6.90)*	4.49*	0.000*
^b^age	−0.27(0.10)*	− 0.12(0.05)*	−2.80*	0.007*
^c^follow-up time	−0.11(0.08)	−0.12(0.09)	−1.39	0.170
^d^waist	−0.22(0.10)*	−0.09(0.04)*	−2.29*	0.026*
^e^VO2-max	0.32(0.11)*	0.19(0.06)*	2.99*	0.004*
^f^sum3bdfg	0.26(0.09)*	0.69(0.24)*	2.84*	0.006*

*n* = 64 (Casewise deletion of missing data, total group of 101 female patients)

***R***^**2**^ **= 0.620**

Variables: ^a^MSH i.e. maximal step-up height the mean of right and left leg (cm), ^b^age (years), ^c^follow-up time i.e. time from baseline to 14–30-month (mean 22) follow-up (months), ^d^waist circumference (cm), co linearity to BMI and weight, ^e^VO2-max i.e. maximal oxygen uptake (L•min-1), and ^f^sum3bdfg denotes any limitations in the SF-36 scale physical function in items 3b, 3d, 3f and 3 g. Standard error = SE. * Denotes p-level < 0.05

**Table 6:** Regression model with mean change of maximal step-up height (^a^MSH) as dependent variable

Variables	Beta (SE)	B (SE)	t (57)	p-level
intercept		6.58(2.24)*	2.94*	0.005*
mean MSH at baseline	−0.17(0.11)	− 0.10(0.07)	−1.55	0.127
^b^follow-up time	−0.42(0.10)*	−0.28(0.09)*	−4.07*	0.000*
^c^BMI change	−0.29(0.11)*	−0.39(0.15)*	−2.58*	0.012*
^d^VO2-max change	0.16(0.11)	0.08(0.06)	1.46	0.149

*n* = 62 (Casewise deletion of missing data, total group of 101 female patients)

***R***^**2**^ **= 0.426**

Variables: ^a^MSH i.e. maximal step-up height the mean of right and left leg (cm), ^b^follow-up i.e. time from baseline to 14–30-month (mean 22) follow-up (months), ^c^BMI i.e. body mass index, with collinearity to waist and weight, ^d^VO2 max i.e. maximal oxygen uptake (L•min-1). Standard error = SE. * Denotes p-level < 0.05

As demonstrated in Table 5, MSH is affected by age and metabolic factors, but not by length of time to follow-up. This result contrasts with the change in MSH (Table 6) where the length of time to follow-up has the largest correlation to MSH maintenance, together with the change in BMI.

Logistic regression was performed with maintained MSH as outcome, and in a univariate model the number of mixed aerobic and strength sessions was significant, OR 3.33 (95% CI 1.25–8.89). In the final multivariate model (*n* = 98) this factor was no longer significant, OR 1.95 (95% CI 0.58–6.61). Covariates were the length of time between the first follow-up (T1) and long-term follow-up (T2) OR 0.78 (95% CI 0.68–0.90), MSH at baseline OR 0.88 (95% CI 0.78–1.00, *p* = 0.043), and age at baseline OR 0.92 (95% CI 0.87–0.99). Goodness-of-fit by Pearson’s test was 0.20, and by Hosmer-Lemeshow’s test was 0.13.

## Discussion

### Main findings

Our main findings were that a primary care-based 3-month group exercise intervention increased leg-muscle strength and function assessed as maximal step-up height (MSH). Furthermore, we found that MSH was declining to below baseline level at 14–30 months follow-up. By that time, our female patients had stopped with their regular group training 2–3 times per week, but reported brisk walking most days of the week as their main exercise. Furthermore, at the long-term follow-up, at a mean of 22 months, the patients still had remaining positive effects on most outcomes. Moreover, at the first follow-up directly after the 3-month period of group exercise as part of the intervention program, patients had a significant decline in weight and BMI. Weight stability compared to baseline remained at the long-term follow-up. Clinically acceptable inter-examiner variation at 4 cm found in this study is a minor concern compared to test-retest variation, which measures at 6 cm [27]. This means that to achieve high validity of MSH results within a study, it is more important that examiners are robust in their testing methods than that several different examiners are used. Furthermore, the inter-examiner repeatability was tested at long-time follow-up to mimic future clinical situations with repeated testing, and with patients with different degrees of physical fitness.

Group exercise included different workouts, mostly mixed aerobic fitness and strength training with a moderate to high intensity level 13–15 on 6–20 Borg RPE scale, which the patients trained to use for intensity control. However, also sessions with merely strength, balance and coordination training were offered to the patients, see Additional file 1.

Analyzing MSH subgroups according to level of maintenance of MSH at long-term follow-up, we found that even the subgroup with the lowest maintenance of MSH maintained their MSH at the follow-up directly after the 3-month group-training period. Thereafter their MSH declined from different MSH baseline levels (Fig. 1a and b), and did so more steeply than for participants in the other subgroups. Most important for MSH decline—in a logistic regression multivariate analysis with maintenance of MSH as outcome—was the length of time before long-term follow-up, indicating a continuous decline of leg-muscle strength and function detectable within a mean period of 22 months. Secondly, an increase in BMI and thereafter higher age (increased sarcopenia) and higher MSH levels at baseline were important for MSH decline.

In the subgroup with the lowest maintenance of MSH, 44% of the female patients had been put on sick leave by the time of the long-term follow-up, compared to 27% of the patients with the highest maintenance of MSH. Changes in MSH and increased sedentary behavior (SB)—due to reduced working capacity and cessation of transportation to work—are further discussed in Additional file 3, section I. These could be reasons for steeper MSH decline in our study but SB was not assessed. Furthermore, evidence has been presented in a recent meta-analysis in which lifestyle interventions showed the potential to reduce SB in adults [38].

Thus, those who during holiday season—the three summer months—could find regular group exercise with mixed aerobic fitness and strength training, increased their ability to maintain leg-muscle strength and function assessed as MSH. Brisk walking most days of the week was not enough to maintain these individuals’ leg strength and function. Also, lower levels of support from primary health care professionals after the program to encourage patients to prioritize regular group exercise—with mixed aerobic fitness and strength training 2–3 times per week with the subjective intensity of 13–15 on the 6–20 Borg scale—could be a reason for the steeper MSH decline beyond the expected due to age.

### MSH along a time axis visualizes changes in muscle strength

Patients with high baseline MSH appeared more frequently early on the time axis in the on-going intervention project (see Fig. 1a). One important reason for this was that patients with many diseases and consequently lower MSH were deemed not to manage the training intervention by their GP, for both physical and mental reasons. The opinions of GPs, however, changed over time after positive reports from patients. According to a recent European multicentre study paper, the greatest difference in mortality risk was observed between the two lowest activity groups for a population with abdominal and general adiposity, as in our female patient population [39].

### Mixed aerobic fitness and strength training with higher intensity important for MSH

In a meta-analysis, combined aerobic and resistance training—the most effective training modality to reduce anthropometric outcomes—have been recommended in the prevention and treatment of overweight and obesity whenever possible [40]. Mixed aerobic fitness and strength training 2–3 times per week in our study (see Additional file 1) could significantly improve MSH and VO_2_-max at both follow-ups and also waist circumference at long-term follow-up. Brisk walking—as reported in our female patient population as the main exercise at long-term follow-up with the mean of 22 months—was at the level of the recommended 150 min per week with moderate activity [2], but this was not enough for maintenance of their MSH. In a systematic review, interventions increased walking among targeted participants on an average of up to 30–60 min per week, at least in the short term. Important for a successful increase was when intervention was tailored to participants’ needs and targeted at those with low physical activity [41]. The group exercise intervention in this study was designed with the same intentions and could be recommended for women with common diseases, and with low working capacity, as well as to patients with cardio-metabolic risks.

### Women in their 50s and cardio-metabolic risk

It is known that cardiorespiratory fitness in general declines at a nonlinear rate which accelerates after 45 years of age [42], and possibly the situation is the same for MSH. However, the women patients in their 50s (Table 3; 3c) in our study had lower aerobic fitness compared to the oldest age group, see Additional file 3, section II.

Another notable lifestyle change was that eleven out of twenty smokers had stopped smoking at long-term follow-up.

### Change in MSH and metabolic risk

The subgroup with highest maintenance of MSH at long-term follow-up had lower mean MSH (cm) (SD) at baseline, 26.7 (4.1) compared to 29.6 (6.0) in the group with the lowest maintenance of MSH, see Additional file 3, section III. No significant difference was seen in mean levels of sick leave between groups at baseline. When a person with overweight changes lifestyle in a short time—for example, being put on sick leave and becoming less physically active without simultaneous weight loss—the decline in leg-muscle strength gives reduced ability to move the body vertically as assessed with the MST. Change in MSH indicate metabolic risk. Also, with rapid reduction in muscle strength and function and no change in energy intake, an increased weight may start a negative spiral towards an even higher BMI, leading to further decline in MSH.

### Change in MSH and obesity and type 2 diabetes

Our data presented in Additional file 2 shows that patients with obesity (BMI ≥ 30) had a higher MSH at baseline than the group with overweight and normal BMI and that the body height did not differ between these groups. Furthermore, the obese patients showed less improvement of MSH during intervention and a steeper decline at long-term follow-up. More information about baseline MSH and the MSH changes in different subgroups are presented in Additional files 2 and 3.

### Change in SF-36 scores and SF-36 physical function items

In addition to patients’ increased metabolic and cardiovascular risks, mentioned above, lower self-reported quality of life in itself is associated with an increased risk of ill health. Comparing our patients’ mean SF-36 scores (broad standard deviations) in Table 2, the eight scales at baseline, with normative Swedish female population scores (narrow confidence interval) with women in the age groups in question of 45–54 years [43] the differences in scores are PF 19, RP 49, BP 31, GH 30, VT 32, SF 22, RE 33 and MH 16. Significantly improved values compared to baseline were in our study measured at both follow-ups (Table 2) and we suggest that an intervention of this kind should be further investigated in a larger context.

We analysed in more detail some questions in SF-36, PF which measures the ability of completing activities of moderate strain level where leg strength might have importance to the estimated function (Table 2). The ability to walk more than 2 km at a time without strain was the easiest task to achieve and keep until the long-term follow-up, but also those who improved their ability to escalate several stairs without large or small difficulty could maintain this ability long term. One third of the patients with reported large problems, mostly with knee bending, reported decreased limitations at the 3-month follow-up shortly after the period of group training. At long-term follow-up the positive effect on knee function had returned to baseline, indicating the need for regular mixed aerobic fitness and strength training. We conclude that the SF-36 items 3b, 3d, 3f and 3 g are useful in clinical practice, both for screening of leg muscle strength and function, to enhance individually prescribed training, and be a help for setting goals.

### Strengths and limitations of the study

The study population was representative of female patients in Swedish primary health care. The intervention program was received well by patients, despite substantial burdens of disease. There were no accidents requiring medical attention during or after the training sessions. All training sessions, except Nordic Walking, included music and in a recent study this was recommended to promote PA among sedentary individuals [44]. The baseline and 3-month follow-up tests were distributed across three out of the four seasons because of the way in which patients were added to the program, and no seasonal influence was discovered. One limitation is that we have not measured SB or PA objectively, including measuring intensity with an accelerometer. Such measurements may have identified additional factors for maintaining MSH. Another limitation is that when presenting the long-term results from subgroups, each subgroup is comparatively small. Because this study is the first to describe long-term results of MSH change after an intervention has been completed, we believe that our results are valuable for planning future intervention studies, see Additional file 2. Another limitation is that the study did not investigate its findings in a population of men.

### Advantages and possibilities with the maximal step-up test

For the diagnosis of sarcopenia, a consensus report recommends the use of the presence of both low muscle mass and low muscle function (strength or performance) [45]. An algorithm recommended for sarcopenia in older individuals includes measurements of gait speed, grip strength and muscle mass. A recently published study showed that handgrip strength may not be an appropriate surrogate for lower-body strength, power or balance, and is proposed only to be used describing upper-body strength or functionality [46]. MST is a weight-bearing test that in an integrated and functional way assesses leg-muscle strength, power, mobility, balance and coordination and is also indirectly a test of metabolic and cardiovascular risk as measuring the function of the body’s largest tissue with secretory capacity—the skeletal muscle.

Exercise has been recommended as a first-line treatment of degenerative joint disease of the hip and knee. The 30-s chair-stand test, 40 m fast-paced walk test and a stair-climb test were recently recommended as the minimal core set of performance-based tests for hip or knee osteoarthritis research and in clinical practice [29, 47]. New research recommends that exercise should more closely target the sensorimotor deficiencies and functional instability associated with the degenerative joint disease of the knee than in traditionally used training methods [48]. MST has been proposed as a recommended performance-based test to assess leg strength and function and side difference in people diagnosed with knee osteoarthritis [12]. Muscle strength, power and joint mobility are perishable, which is why repeated testing is needed, especially in patients with cardiovascular and metabolic diseases and symptomatic degenerative joint disease of the knee and hip, see also Additional file 3, section IV.

## Conclusions

A 3-month group exercise intervention significantly improved maximal step-up height, aerobic fitness and self-reported health, and decreased risk in female patients with elevated cardio-metabolic risk and low self-reported health. At an average of 22-month follow-up investigation, when brisk walking was reported as the main exercise, the maximal step-up height was reduced to lower than baseline. However, significant positive effects remained for waist circumference, VO_2_-max, self-reported health, physical function as well as exercise and physical activity. Regular group exercise 2–3 times per week with mixed aerobic fitness and strength training, and with support from health care professionals, is probably needed to maintain improved leg-muscle strength and function among these primary care patients.

## Supplementary information

**Additional file 1.** Mean (SD) participation rate during and after a 3-month group exercise intervention in female patients.

**Additional file 2.** Baseline levels and changes (mean (SD)) to follow-ups sorted as total group and subgroups.

**Additional file 3.** The usefulness of maximal step-up test in clinical everyday work.

## Data Availability

The datasets generated and analyzed during the current study are not publicly available as it is a clinical study on patients, but are available from the investigator/corresponding author Dr. Lillemor Nyberg (lillemor.nyberg@oru.se) on reasonable request for research. The dataset in Excel contains a description of patients, baseline measurements and at 3 and 22 months follow-up of primary and secondary variables. This data will be available after publication without time limit. Data anonymization, no other restrictions.

## Abbreviations

Biodex: Isokinetic strength test
BMI: Body mass index
Borg RPE Scale: Rating of perceived exertion
MSH: Maximal step-up height
MST: Maximal step-up test
Nordic Walking: Walking with rods
OR: Odds ratio
PA: Physical activity
PF: Physical function
SD: Standard deviation
SF-36: Self-reported health
T0: Baseline measurements
T1: Measurements after 3 months
T2: Measurements after 14–30 (mean 22) months
VO_2_-max: Maximal oxygen consumption
