# Supplementary material for: Long-term effects of group exercise intervention on maximal step-up height in middle-aged female primary care patients with obesity and other cardio-metabolic risk factors
Source: BMC Sports Sci Med Rehabil. 2020 Mar 16;12:11. doi: 10.1186/s13102-020-00161-4 (PMC7074992; doi:10.1186/s13102-020-00161-4)
Supplement: Supplementary file 1 — Additional file 1. Mean (SD) participation rate during and after a 3-month group exercise intervention in female patients. [file 13102_2020_161_MOESM1_ESM.pdf]

Additional file 1: Mean (SD) participation rate during and after a 3-month group exercise intervention in female patients.

Variable	T0–T1(n)	number(SD)	–T2(n)	number(SD)
[1]**Group sessions—total number (SD)**	98	27(8)	51	15(15)
mixed aerobic and strength	95	16(7)	29	12(16)
mostly aerobic	59	12(6)	35	9(8)
mostly strength	48	9(5)	13	10(8)
[2]balance, coordination	32	10(4)		
[3]**Total number of sessions**			97	62(48)
mostly brisk walking			95	48(35)

[4]**Group sessions—per week**	**T0–T1(n)**	**t.p.w.**	**m.p.w**	
	80	2.5	150	
	15	1.4	85	
	3	0.8	50	

[5]**Group sessions—sorted by:**	**T0–T1(n)**	**t.p.w.**	**m.p.w**	
mixed aerobic and strength	1	2.8	170	
	23	1.9	115	
	48	1.3	75	
	23	0.6	40	
mostly aerobic	5	2.1	130	
	23	1.1	70	
	31	0.6	40	
mostly strength	1	2.7	160	
	14	1.1	65	
	33	0.6	35	
balance, coordination	32	0.8	50	

Time points: T0–T1 = follow-up assessment after 3 months with mostly mixed aerobic fitness and strength training in a lifestyle and group exercise intervention program including 101 female primary care patients, –**T2 =** at 14–30 months follow-up, the assessment of self-reported exercise/physical activity during previous 3 months registered from diaries, mostly brisk walking. **n** = number of patients. **number** = amount of sessions. [1]The type and dose of group training sessions with the objective intensity 13–15 (6–20 Borg RPE Scale) during 3-month intervention program (T0–T1). [2]Dance and movement therapy numbers also registered

and included for analysis as mostly aerobic, and Qigong as mostly strength. [3]The total number of sessions *i.e.* total sum of group exercise together with individual training mostly aerobic *i.e.* brisk walks ≥ 30 minutes at 11–13 (6-20 Borg RPE Scale). [4]Supervised group exercise sessions total amount, and [5]Group exercise sorted according of the type of exercise for the total patient population and presented as times per week (t.p.w) or minutes per week (m.p.w).
