# Supplementary material for: Long-term effects of group exercise intervention on maximal step-up height in middle-aged female primary care patients with obesity and other cardio-metabolic risk factors
Source: BMC Sports Sci Med Rehabil. 2020 Mar 16;12:11. doi: 10.1186/s13102-020-00161-4 (PMC7074992; doi:10.1186/s13102-020-00161-4)
Supplement: Supplementary file 2 — Additional file 2. Baseline levels and changes (mean (SD)) to follow-ups sorted as total group and subgroups. [file 13102_2020_161_MOESM2_ESM.pdf]

Additional file 2: Baseline levels and changes (mean (SD)) to follow-ups sorted as total group and subgroups.

Variables	n	T0	n	T0–T1	n	T0–T2
[1]**All patients with MSH**	98	27.2(5.7)	85	1.5(2.2)	89	-2.4(3.0)
**Subgroups:**						
[2]**Intensity T0–T1 (Borg)**						
moderate (13-15)	31	29.5(4.8)	29	2.4(2.3)	30	-1.9(3.6)
light (11-13)	51	26.7(5.6)	45	1.1(1.8)	45	-2.3(2.79
light-light (9-11)	13	23.5(6.8)	11	0.7(2.5)	12	-3.6(2.4)
[3]**Knee or Hip at T0**						
not knee or hip	57	27.6(5.9)	52	1.4(2.0)	52	-2.4(2.7)
just knee	15	27.9(4.6)	14	1.9(2.7)	14	-1.1(4.0)
both knee and hip	12	26.5(4.8)	11	2.1(2.4)	12	-1.9(1.7)
just hip	14	25.6(7.0)	8	0.4(1.8)	11	-4.6(3.2)
[4]**BMI at T0**						
BMI < 25, no T2D	24	28.2(5.9)	22	1.9(2.0)	22	-2.1(2.8)
BMI < 25, T2D	2	15.0(4.2)	1	3.0(0.0)	1	0.0(0.0)
BMI 25-<30, no T2D	24	27.1(5.6)	20	1.9(2.7)	20	-1.4(3.5)
BMI 25-<30, T2D	3	25.0(2.3)	1	1.5(0.0)	2	-4.5(4.2)
BMI 30-, no T2D	34	28.5(4.7)	31	1.0(2.1)	33	-2.7(2.7)
BMI 30-, T2D	10	23.4(6.2)	9	0.8(1.5)	10	-4.1(3.1)
[5]**Neck or Shoulder at T0**						
no pain in neck or shoulder	27	25.7(6.4)	23	1.3(1.7)	24	-2.9(3.0)
neck	6	26.8(2.4)	5	2.1(2.5)	5	-1.8(1.3)
shoulder	11	27.8(3.9)	10	1.1(3.5)	10	-2.3(4.5)
neck and shoulder	54	27,9(5.9)	47	1.6(2.1)	50	-2.2(2.9)
[6]**Mental disorder at T0**						
a	56	26.1(6.5)	49	1.5(2.3)	50	-2.5(3.4)
b	15	29.2(3.9)	13	1.2(1.6)	13	-1.7(2.9)
c	14	28.2(4.8)	11	0.8(1.7)	14	-3.0(2.3)

d	3	30.5(3.8)	3	0.0(0.0)	3	-3.0(3.0)
e	10	28.2(4.2)	9	3.2(2.3)	9	-1.5(2.3)

Tests analyzing significant differences between subgroups or changes at follow-ups were not conducted due to small numbers. [1]All patients with baseline MSH *i.e.* total group of 101 female patients. [2]Intensity 6-20 Borg RPE Scale; lowest level during supervised sessions. [3]Knee and or hip pain and reduced function. [4]BMI *i.e.* body mass index; T2D *i.e.* diabetes type 2. [5]Neck and or shoulder pain and reduced function. [6]Mental disorder at T0; a = no mental disorder, b = stress + at least one other (not depression), c = depression + at least one other (not stress), d = stress + depression + at least one other, e = only at least one other (not depression or stress) *i.e.* sleep disturbance, anxiety, fatigue and/or fibromyalgia.
