# Supplementary material for: Long-term effects of group exercise intervention on maximal step-up height in middle-aged female primary care patients with obesity and other cardio-metabolic risk factors
Source: BMC Sports Sci Med Rehabil. 2020 Mar 16;12:11. doi: 10.1186/s13102-020-00161-4 (PMC7074992; doi:10.1186/s13102-020-00161-4)
Supplement: Supplementary file 3 — Additional file 3. The usefulness of maximal step-up test in clinical everyday work. [file 13102_2020_161_MOESM3_ESM.pdf]

Additional file 3

I. Sedentary behavior, obesity, changes in MSH and reduced capacity to work

In the subgroup with the lowest maintenance of MSH, 44% of the female patients had been put on sick leave by the time of the long-term follow-up, compared to 27% of the patients with the highest maintenance of MSH. The reasons for decline in MSH may be extended absence from work and transportation to work, and not participating in any mixed aerobic fitness and strength training, or the fact that just one session per week was not enough to maintain MSH. Also, many participants in the total group were overweight or obese at the start of the intervention. Notable was that the obese patients had the highest mean MSH of both legs at baseline. At long-term follow-up, without reported regular group exercise, leg-muscle strength to maintain the ability to move the body vertically in MST was not enough. It was hardest for the obese patients to maintain MSH. If there had been significant statistical power when doing logistic regression analysis, mixed aerobic fitness and strength training would probably have had significant positive association with maintenance of MSH. Sickness and obesity, together with reduced capacity to work and insufficient daily PA, appear in our study to create this MSH decline earlier than muscle weakness due to age would have resulted in [1, 2], and with an increased cardio-metabolic risk. Higher BMI as determinant of decreased activity has previously been reported [3]. In occupational health when preventing obesity and weight gain, early support measures have been recommended for obese employees in the form of a focus on enhanced physical functioning, and treatment of obesity and its co-morbidities [4].

Analysing MSH change over time shows that a new functional status can appear quite fast related to decreased muscle strength. When changing to a more inactive lifestyle—for example, when being put on sick leave, getting sickness benefits or being unemployed—with

a rapid decline in leg muscle strength and leg function as a result, the metabolic and cardiovascular risks are markedly enhanced [5].

II. Patients in their 50s and cardio-metabolic risk

When divided in tertiles by age, unexpectedly the women patients in their 50s (table 2; 3c), had lower aerobic fitness levels compared to the oldest age group. Also, their MSH level and waist circumference were in between the levels in the other groups but closer to the lower level of the oldest age group than to that of the youngest group. It is known that cardiorespiratory fitness in general declines at a nonlinear rate which accelerates after 45 years of age [6], and possibly the situation is the same for MSH. Furthermore, it has been shown that elderly women with metabolic syndrome have lower functional capacity, muscle strength, lower limb power and flexibility when compared to women without metabolic syndrome [7]. With high waist circumference, low aerobic fitness and relatively low leg-muscle strength, metabolic syndrome is a probable diagnosis for many of our patients in their 50s.

III. Sickness benefits, change in MSH and metabolic risk

On the one hand, at baseline, the group with the highest maintenance of MSH compared to that with the lowest, included some patients on sickness benefits or with disability pensions, which may be a possible cause of lower MSH. The group with the lowest maintenance at long-term follow-up, when one fourth of the patients received either sickness benefits or disability pension, and nearly half (44%, n=22) compared to one third (27%, n=27) of patients in the group with the highest maintenance, had been put on sick leave. Accordingly, the MSH decline we found in our study at long-term follow-up can be an early finding indicating

effects of sick leave and lower leg-muscle strength on the potential for increased cardio-metabolic and functional risks.

IV. Maximal step-up test – a health check in both clinical practice and as a self-test

A steep decline in MSH was seen for patients with type 2 diabetes indicating loss of muscle strength and function and comparative reduction in metabolic capacity. In attempting to find an assessment method for detecting unhealthy obesity, i.e. obesity with low leg-muscle strength and metabolic function, the MST assessment of MSH could be considered useful when planning for future research.

MST can be used for future research to study: the natural course of sarcopenia and osteoarthritis; the influence of exercise interventions on the risk of falls; the morbidity and mortality in cardio-metabolic diseases, COPD and several other chronic diseases [8, 9].

The measurement of MSH has many advantages, already presented [10, 11], it is easy and safe to perform and immediately delivers a measurement value. Furthermore, it is inexpensive, and after brief instruction, MST can be performed by healthcare staff with the help of step-up boards with different heights. Potentially, MST could, in everyday clinical practice on consecutive patients, allow the clinician and nurse to detect low muscle strength and abnormalities in the loco motor system not observable with the patient in a resting position. A review has recently provided evidence that suggests aerobic exercise assessment to be a vital sign status in future clinical practice [12]. After the same considerations about the health effects and usefulness of assessment of leg strength, we suggest MST may serve as a vital sign status for assessment of muscle strength and function which could be used—as a health check—in both clinical practice and as a self-test.

REFERENCES

1. Cederholm T, Morley JE: **Sarcopenia: the new definitions**. *Curr Opin Clin Nutr Metab Care* 2015, **18**(1):1-4.

2. Cruz-Jentoft AJ, Landi F, Topinkova E, Michel JP: **Understanding sarcopenia as a geriatric syndrome**. *Current opinion in clinical nutrition and metabolic care* 2010, **13**(1):1-7.

3. Golubic R, Wijndaele K, Sharp SJ, Simmons RK, Griffin SJ, Wareham NJ, Ekelund U, Brage S: **Physical activity, sedentary time and gain in overall and central body fat: 7-year follow-up of the ProActive trial cohort**. *Int J Obes (Lond)* 2015, **39**(1):142-148.

4. Roos E, Laaksonen M, Rahkonen O, Lahelma E, Lallukka T: **Weight change and sickness absence-a prospective study among middle-aged employees**. *Eur J Public Health* 2015, **25**(2):263-267.

5. Wennberg P, Lindahl B, Hallmans G, Messner T, Weinehall L, Johansson L, Boman K, Jansson JH: **The effects of commuting activity and occupational and leisure time physical activity on risk of myocardial infarction**. *Eur J Cardiovasc Prev Rehabil* 2006, **13**(6):924-930.

6. Jackson AS, Sui X, Hebert JR, Church TS, Blair SN: **Role of lifestyle and aging on the longitudinal change in cardiorespiratory fitness**. *Arch Intern Med* 2009, **169**(19):1781-1787.

7. Vieira DC, Tibana RA, Tajra V, Nascimento Dda C, de Farias DL, Silva Ade O, Teixeira TG, Fonseca RM, de Oliveira RJ, Mendes FA *et al*: **Decreased functional

capacity and muscle strength in elderly women with metabolic syndrome. *Clin Interv Aging* 2013, **8**:1377-1386.

8. Nyberg LA, Hellenius ML, Kowalski J, Wandell P, Andersson P, Sundberg CJ: **Repeatability and validity of a standardised maximal step-up test for leg function-a diagnostic accuracy study**. *BMC Musculoskelet Disord* 2011, **12**:191.

9. Nyberg LA, Hellenius ML, Wandell P, Kowalski J, Sundberg CJ: **Maximal step-up height as a simple and relevant health indicator: a study of leg muscle strength and the associations to age, anthropometric variables, aerobic fitness and physical function**. *British journal of sports medicine* 2013, **47**(15):992-997.

10. Zanotto T, Bergamin M, Roman F, Sieverdes JC, Gobbo S, Zaccaria M, Ermolao A: **Effect of exercise on dual-task and balance on elderly in multiple disease conditions**. *Curr Aging Sci* 2014,**7**(2):115-136.

11. Gobbo S, Bergamin M, Sieverdes JC, Ermolao A, Zaccaria M: **Effects of exercise on dual-task ability and balance in older adults: a systematic review**. *Arch Gerontol Geriatr* 2014, **58**(2):177-187.

12. Arena R, Myers J, Guazzi M: **The future of aerobic exercise testing in clinical practice: is it the ultimate vital sign?** *Future Cardiol* 2010, **6**(3):325-342.
